# Combating healthcare corruption and fraud with improved global health governance

**DOI:** 10.1186/1472-698X-12-23

**Published:** 2012-10-22

**Authors:** Tim K Mackey, Bryan A Liang

**Affiliations:** 1 Institute of Health Law Studies, California Western School of Law, San Diego Center for Patient Safety, University of California San Diego School of Medicine, 350 Cedar Street, San Diego, CA, 92101, USA; 2 Joint Doctoral Program on Global Health, University of California San Diego-San Diego State University, San Diego, CA, USA; 3 San Diego Center for Patient Safety, University of California San Diego School of Medicine, 350 Cedar Street, San Diego, CA, 92101, USA; 4 Department of Anesthesiology, University of California San Diego School of Medicine, San Diego, CA, USA

**Keywords:** Global health, Global health governance, Corruption, Informal economy, International law, Health policy, Health system strengthening

## Abstract

Corruption is a serious threat to global health outcomes, leading to financial waste and adverse health consequences. Yet, forms of corruption impacting global health are endemic worldwide in public and private sectors, and in developed and resource-poor settings alike. Allegations of misuse of funds and fraud in global health initiatives also threaten future investment. Current domestic and sectorial-level responses are fragmented and have been criticized as ineffective. In order to address this issue, we propose a global health governance framework calling for international recognition of “global health corruption” and development of a treaty protocol to combat this crucial issue.

## Correspondence

The impact of globalization on health has been marked by new and daunting challenges. Though globalization has enabled advancements in trade, travel, and communications, it has also facilitated rapid global spread of infectious diseases such as SARS and H1N1/A, requiring a paradigm shift in global health governance. Yet, the next global pandemic is not the only challenge facing global health. A more immediate threat is systemic global healthcare corruption that adversely impacts both developed and resource-poor states.

As reported by Transparency International, the scale and scope of corruption impacting health is immense. Exact numbers are elusive, but it is estimated billions of dollars are lost annually due to corruption and fraud in a global health market estimated to be worth 10% of global gross domestic product in 2009 [1-3]. Systematic corruption in health is also a barrier in meeting the Millennium Development Goals as it weakens health systems and delivery [4]. It also disproportionately impacts the vulnerable, including negative health outcomes for women and children [4].

Importantly, health corruption not only leads to financial waste of scarce resources, but also has adverse impact on healthcare access, infrastructures, financing, and social determinants of health. In addition, health corruption can severely compromise quality and coverage of services, leading to price inflation for health service unit costs [5].

Indeed, health corruption at the domestic level represents a severe impediment to global health efforts in resource-poor settings and transitional economies. Corruption can drain resources from already impoverished and fragile health systems, precluding access to life-saving treatment for vulnerable patient populations [4,6]. With surveys reporting that 80% of individuals in developing countries have experienced health sector corruption, these resource-poor populations are disproportionately impacted [4,7,8].

Recent scandals that have plagued global multilateral health programs have also raised concerns regarding presence of corruption in global health. Allegations of corruption and fraud in the Global Fund to Fight AIDS, Tuberculosis and Malaria (the “Global Fund”) represent a serious threat to continued funding and support of global health initiatives. This is particularly concerning given ongoing funding challenges arising from the recent global economic crisis [9].

The confluence of domestic and international level health corruption impacting global populations points to a lack of an international framework specifically addressing the wide range of interrelated issues associated with health-related corruption. Improved global health governance is an important first step. Reform to detect and eliminate forms of corruption that impact health must be coordinated in order to ensure that health systems are protected and that global health interventions meet their full potential.

### Global Impact of Domestic Health Corruption

Corruption is defined as the “misuse of entrusted power for private gain” [10]. In the health setting, it can encompass bribery of health professionals, regulators and public officials; unethical research; diversion/theft of medicines and medical supplies; fraudulent or overbilling for health services; absenteeism; informal payments; embezzlement; and corruption in health procurement [4,10,11]. Though many of these activities are domestically-focused, they nevertheless impact global health outcomes. Domestic corruption may have global results due to interconnectedness of domestic and global health financing, a globalized drug supply chain, health worker migration, and shared global health security.

In combination with the reality that criminal activities can easily impact and move across geopolitical borders affecting multiple states/organizations, health corruption presents significant challenges for detection and enforcement. The broad infiltration of corruption, diversity of illicit acts, and multitude of stakeholders involved (often with limited or indirect accountability) adds to health system complexity, existing information asymmetry, and uncertainty in health markets, with developing countries particularly vulnerable [4,10].

Beyond developing countries, domestic corruption is also endemic in developed and emerging economies. For example, 5-10% of USA public sector health expenditures are lost to fraudulent overbilling [2]. Recent reports in the USA of $4 billion in recoveries in enforcement actions, a $163 million indictment against an organized crime enterprise for fraudulent billing, and other cases highlight the near universality of domestic level health corruption and fraud [12,13]. As well, 5% of the Cambodian health budget is lost to corruption within the central government, and 56% of Russian Federation total health expenditures are “informal” payments [2].

The global drug supply chain also provides an illustration of how lack of regulatory harmonization can lead to forms of global health-related corruption [14,15]. Health systems in developing regions and emerging markets that lack transparency, regulatory control, and adequate law enforcement can result in public and private sector extortion and bribes that enable production and sale of counterfeit drugs [14,15]. As a result, counterfeit medicines can be exported to other global markets, resulting in documented patient death and morbidity, pathogen resistance and decreasing drug effectiveness, waste in resources for pharmaceutical products and services, and lack of safe access to essential drugs [14-16].

Note that health corruption also extends to legitimate corporate entities. For example, corruption allegations against multinational pharmaceutical and medical device companies such as Johnson & Johnson, Merck, Eli Lilly, and Medtronic have surfaced involving bribery of physicians and health officials overseas. As pharmaceutical companies push to gain entry into emerging markets, competition gives rise to corrupt practices and have been prosecuted as potential violations under USA and UK foreign anti-bribery laws [17]. Reflecting this trend, recently the world’s largest drug manufacturer, Pfizer, agreed to pay a fine of more than $60 million to settle violations of USA anti-bribery laws for improper payments to healthcare professionals in a number of countries including China, Russia, Bulgaria, Croatia, Kazakhstan and Italy [18].

**Figure 1:**
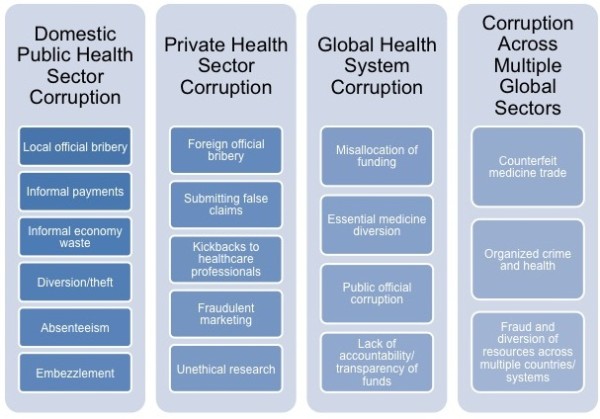
Examples of health corruption types and scopes.

This broad spectrum of domestic level health corruption that has a global impact encompasses countries at all levels of development, spans various types of illicit activities, and includes both public and private actors. (See Figure 1) The broad scope of the problem necessitates development of systems of international cooperation and governance in order to mount a comprehensive solution.

### Corruption in Global Health Interventions

Large scale, multilateral global health programs also present new opportunities for health corruption. Alleged domestic corruption in developing countries receiving aid through the multibillion-dollar Global Fund has recently shaken public trust in global health initiatives and financing [19]. Yet these are not the first allegations of corruption in the Global Fund or other multilateral global health projects.

For example, corruption was discovered in 2008 in five multimillion-dollar health projects financed by the World Bank in India. The program was aimed at combating tuberculosis, malaria and HIV/AIDS. Investigation uncovered procurement corruption, including bid-rigging and bribery [20]. Further, investigators discovered substandard HIV/AIDS testing kits, which may have produced incorrect results and exacerbated disease spread [20]. Yet, Indian public officials have expressed a level of acceptance regarding the corruption allegations, commenting it is systemic in India [20]. India is also a known for having a large informal health sector susceptible to corruption and informal payments for health services [7,20].

Sovereign country representatives are also involved. For example, corruption in Uganda involving Global Fund disbursements of $45.3 million in 2006 revealed illegal acts by public officials, government ministers, and community health workers [21]. Several actors defrauded the Global Fund, leading to the temporary grant suspension and interruption of patient antiretroviral therapy with adverse consequences for its recipients [21]. Corruption was uncovered by a whistleblower, *not* internal evaluators, and later verified by an independent auditor and national judicial commission that detailed misappropriation, forgeries, nepotism, and lack of accountability [21].

Other findings show continued corruption in these initiatives. For example, the Global Fund’s own independent audit unit and later the Associated Press revealed $34 million in Fund grants that may have been misused in Djibouti, Mali, Mauritania, and Zambia [19]. This includes allegations of forged documents, improper bookkeeping, donated drugs diverted and sold on the black market, and a range of 30-67% of funds reportedly misspent [19]. This prompted some donor countries (e.g., Germany, Ireland, Sweden) to announce they would be suspending support of the Global Fund in light of corruption policy concerns [22,23].

Concerns also extend to the U.N. Development Program itself, which manages half the Global Fund’s spending. It refused to share internal audit reports citing its own governance rules on audit controls and disclosure.[19,22] This led to increased scrutiny regarding the integrity of the Fund, further damaging its credibility.

In response, the Global Fund announced it would be restructuring its auditing procedures and establishing additional measures to enhance financial oversight [24]. Coincidentally, the Global Fund recently announced it was suspending future grant funding due to lack of funds following the aftermath of fraud disclosures [25].

The consequences of these allegations make it clear that forms of corruption in global health programs continue to represent a severe risk to progress in these important interventions as donors and the public scrutinize funding use and management. This is despite the fact that the amounts allegedly stolen represent less than 1% of total amounts disbursed by the Global Fund [25].

### Suggested Policy Approaches

In response to the threats posed by forms of domestic and international health corruption, creative global health governance policy solutions should be explored. Individual state-based legal and institutional reform measures that have been proposed include improving allocation, monitoring, auditing and health expenditure tracking; adopting codes of conduct and ethics; and developing rules to increase transparency [5,10]. However, a lack of a comprehensive, internationally cooperative framework specifically addressing health corruption on a global level undermines the effectiveness of these independent efforts.

To address these corruption challenges, we propose a global health governance approach. This would be based upon creating international consensus and recognition of the concept of “global health corruption.” Creating recognition of an overarching definition coupled with development of an internationally binding treaty protocol and a governance framework can lead to cooperation and potential for harmonized laws and regulations to effectively combat global health corruption (see Table 1).

**Table 1:** Key Points of Global Health Anti-Corruption Framework

**GOVERANANCE SYSTEM**	**DESCRIPTION**	**BENEFITS**	**INSTITUTIONAL RESPONSIBILITY**
**Establishing International Consensus on “Global Health Corruption”**	Suggested definition: “misappropriation of authority, resources, trust or power for private or institutional gain that has adverse effects on regional, local, or international health systems and/or that negatively impacts individual patient and/or population health outcomes.”	Establishes an internationally recognized definition and draws needed attention to the unique risks of health-related corruption	International community and input from all relevant stakeholders (e.g. public health agencies, law enforcement, regulators, judicial system, civil society, global health systems, donors)
**WHO-UNODC Global Health Corruption Protocol Under UNCAC**	Development of an international binding treaty protocol on global health corruption and establishing the necessary global health governance framework	Implements definition under an existing international treaty and establishes infrastructure for global corruption framework	Member states of WHO and UNODC
**Global Health Anti-Corruption Governance Framework**	*Model Acts System:* Development of Model Acts system of core anti-corruption definitions and requirements for individual states to implement with certain flexibilities	Development of a model system for states to follow in developing their own domestic systems and aids in harmonization	Signatories to Protocol in consultation with domestic stakeholders
	*Domestic and Regional Corruption Tools:* Assessment of inclusion of existing domestic anti-corruption tools that have had success	Examines existing enforcement tools that have curbed domestic level health corruption	Governance structure of protocol (e.g. conference of state parties, other developed governing body)
	*Useful International Tools and Systems:* Assessment and active inclusion of existing methods, tools and good practices addressing corruption developed by international organizations	Assesses existing tools developed by international organizations aimed at addressing global health system corruption	Governance structure of protocol (e.g. conference of state parties, other developed governing body)
	*Governance System:* Development and implementation of dynamic global health governance structure to address global health corruption flexible enough to deal with diverse forms of corruption in different settings	Governance system flexible enough to be tailored to domestic and global health system needs. Should be comprehensive including components of protocol implementation, financing, incorporation of health system strengthening, and establishment/recommendation of various anti-corruption interventions.	All stakeholders

### A Global Health Corruption Governance Framework

#### Definition of “Global Health Corruption”

A crucial first step in addressing global health corruption is establishing international recognition and consensus of this specific type of corruption while emphasizing the importance of developing anti-corruption tools in health policy and capacity building goals [4]. Though the general concept of “corruption” has been recognized in the U.N. Office on Drugs and Crime’s (“UNODC”) 2003 U.N. Convention against Corruption (“UNCAC”), a legally binding anti-corruption instrument, unique challenges created by both domestic and global health system corruption impacting population-based health have not been delineated [26].

In response, the concept of global health corruption should be expressly defined, discussed, and adopted through an international legally binding framework employing partnership with the World Health Organization (WHO) and the UNODC. This can be accomplished through consultative meetings and development of a Global Health Corruption Treaty Protocol to the UNCAC, with ongoing and active participation of WHO to add provisions specifically addressing health. We believe an effective working definition of global health corruption could start as the: “misappropriation of authority, resources, trust or power for private or institutional gain that has adverse effects on regional, local, or international health systems and/or that negatively impacts individual patient and/or population health outcomes.” This definition should include the varying forms of corruption occurring both at the domestic and global program system level, as both forms of corruption can have an impact on global health.

This standard definition can provide the basis for strategic efforts to coordinate cross-border, regional and international actors to detect and prosecute criminals in concert with existing or future domestic and regional laws that recognize the important differentiation of general corruption and corruption impacting health. This can result in individual countries enacting domestic laws that are harmonized internationally to allow law enforcement to coordinate global anti-corruption strategies for more effective prosecution. This can lead to development of best practices, with defined subsets of forms of global health corruption, identification of common vulnerabilities in health corruption, development of efficient financing mechanisms and judicial systems, and minimum requirements for transparency, enforcement and prosecution.

#### Model Acts System

To implement this governance system, a “model act” approach, similar to USA that allows adoption of model clauses and harmonization by individual states, may be an efficient strategy. This would allow WHO-UNODC to build on agreed upon global health corruption definitions but tailor them for cultural and infrastructural differences, allowing more effective adoption by individual countries sensitive to the local environment. Components could include model requirements for public and private sector policies on disclosure, auditing, procurement, drug supply, and payments to healthcare professionals.

#### Domestic and Regional Corruption Tools

Developed country anti-bribery laws may also serve as potential enforcement tools that can be assessed for inclusion in a global health corruption framework. Laws such as the USA Foreign Corrupt Practices Act and UK Anti-Bribery Act prohibit corporations and individuals based in these countries from bribing public officials in other countries and engaging in other forms of corruption; they have been successful in at least some health sector prosecutions [17,18].

Other national laws, such as the USA Anti-kickback Statute and the False Claims Act, impose both civil and criminal fines for the submission of false claims and for providing forms of remuneration to healthcare professionals for referrals for services covered under national healthcare programs [27]. These laws employ incentive and payment schemes that reward whistleblowers who report illegal activity and have resulted in record-breaking fines against criminal and industry actors [27]. Such enforcement tools could be incorporated into other national healthcare programs to improve enforcement and prevention, and modified so that recovered amounts are earmarked for public health financing/system strengthening. Collectively, these domestic laws currently rely upon developed country enforcement and could be more effective if they had global or regional coverage.

Further, domestic and regional efforts such as the UK’s National Health System anti-fraud unit and the European Healthcare Fraud and Corruption Network could also be considered. These represent models for tailored anti-global health corruption systems that may be scaled up for increased more regional/global coverage.

#### Useful International Tools and Systems

As part of enhanced governance efforts, WHO-UNODC should utilize existing research and guidance on corruption in health, including methods, tools and good practices as developed by the United Nations Development Programme (“UNDP”) [4]. These strategies, include an emphasis on prevention, broad-based partnership and participation with various stakeholders and sectors, whistleblowing mechanisms, and creating incentives and disincentives for good and bad behavior respectively [4]. These efforts can include integration of WHO’s own programs on corruption, including diagnostic and risk assessment tools and the WHO Good Governance for Medicines Programme [4]. They should also integrate World Bank tools such as the Detailed Implementation Review, a proactive diagnostic tool used by the Bank to evaluate projects for indicators of fraud and corruption using forensic accounting and fraud investigation techniques that assess and detect the risk of fraud and corruption. These tools, along with front-line tools developed by USAID, specifically target multilateral global health programs and should be incorporated [4,28]. By coordinating these divergent efforts into a central framework, better policy coherence and cooperation can occur across agencies/platforms.

#### Governance Systems

Recognizing that there is no “one size fits all” approach in establishing a comprehensive framework to deal with all forms of global health corruption, careful attention should be given to programmatic differences between domestic versus global health systems. Hence, vertically integrated global health initiatives spanning the scope of multiple, international stakeholders focused on individual diseases need to be addressed uniquely within the context of susceptibility to corruption. Similarly closed national health systems that are not primarily funded by international health or development assistance and are primary-care focused need to be assessed differently from their global health system counterparts.

Fortunately, global governance models are available for guidance. Using the UNCAC experience, global governance approaches to corruption can include WHO-UNODC member creation of formal and binding systems. This includes establishing a strategy for global health corruption protocol implementation, anti-corruption system financing, and overall health system strengthening. This can be accomplished by providing technical assistance to member states, developing model policies and tools for anti-corruption, identifying “at-risk” countries, exploring innovative financing mechanisms (e.g., funding through allocation of a percentage of anti-corruption fines/recoveries) and coordinating efforts with other multilateral and bilateral development, health, and enforcement agencies, civil society, and the private sector [29]. These efforts can also be coupled with international initiatives to improve aid effectiveness, by integrating developed anti-corruption strategies into efforts of the Paris Declaration on Aid Effectiveness and related forums.

At a minimum, these governance systems should involve multiple interventions and include: (1) transparency and audit policies; (2) a common framework for corruption monitoring and evaluation of public health programs and funding; (3) Codes of Conduct for public and private sector actors; (4) minimum standards for member state laws to specifically prevent and prosecute health-based corruption; (5) health financing improvements to curtail the need for an informal health sector; (6) a centralized surveillance and data repository system to report and investigate global health corruption; (7) multilateral processes to freeze proceeds from corruption and aid in recovery of diverted assets; and (8) commitment to earmark portions of seized assets to fund and develop these anti-corruption systems among members.

This dynamic and comprehensive “global health anti-corruption” framework can build on existing and future best practices, as well as examine previous corruption case studies to determine appropriate strategies that are sensitive to domestic or regional limitations. Though these policies may results in some additional costs and require administrative resources to implement, they are nevertheless crucial in protecting and obtaining the benefits from public health investments, enhancing health system strengthening, and maintaining public trust in global health interventions.

#### Potential Benefits

The potential benefits of the proposed global health governance framework could be significant, starting with global recognition regarding the unique risks of global health corruption and its adverse impact on societies worldwide.

Importantly, this strategy can assist in addressing corruption both on a global and domestic level. Beyond coordinating domestic and international law enforcement efforts, more focused strategies to prioritize rapid review of health projects and systems that are most susceptible to corruption and have the greatest potential negative impact on health outcomes should be addressed immediately [4]. As an example, forms of pharmaceutical sector corruption that enable production and consumption of dangerous counterfeit medicines and represent an immediate patient safety threat can be prioritized in global anti-corruption efforts.

In addition, anti-corruption actions can be strategically targeted for diverse domestic populations by disseminating community-based monitoring tools addressing different forms of health corruption endemic in particular setting (e.g., border and migrant health). As well, proactive regional risk assessment can allocate resources to populations most affected by global health corruption. Such a “top down/bottom up” approach can allow for flexibility of a dual global and domestic approach to combating global health corruption, ensuring better accountability, transparency, and importantly, effective use of anti-corruption investment.

### Summary

Global health corruption remains a serious, ongoing, and under recognized threat to global health progress. Unfortunately, the world’s most vulnerable shoulder much of this life-and-death burden. Further, recent global health program scandals serve to emphasize the need for progressive and global reform.

Under Article 12 of the International Covenant on Economic, Social, and Cultural Rights, every human has a fundamental right to the highest attainable standard of health [30]. Global health corruption undermines this fundamental right. Domestic, international, public and private entities must join together to ensure that health equity is given true weight. This requires a commitment to collectively address global health corruption so that benefits of global health efforts inure to the populations they are intended for.

## Competing interest

The authors declare no potential conflicts of interest or competing interests associated with this manuscript.

## Authors’ contributions

We note that with respect to author contributions, Tim Mackey (TM) and Bryan A. Liang (BAL) jointly conceived the study, TM and BAL jointly wrote the manuscript, TM, and BAL jointly edited the manuscript, and BAL supervised its legal and policy analysis. All authors read and approved the final manuscript.

## Author information

**Tim K**. **Mackey**, **MAS**: TKM is a Senior Research Associate, Institute of Health Law Studies, Ph.D Candidate in the Joint Doctoral Program in Global Health at University of California, San Diego-San Diego State University, and Clinical Instructor at University of California, San Diego. He holds a Masters Degree in Health Law, UCSD-California Western School of Law. He has a diverse professional background including public and private sector experience and completing an internship at the World Health Organization. He has co-authored over 60 publications and given numerous presentation on issues including counterfeit medicines, global health governance, health migration, biodiversity, eHealth, organized crime and corruption, and pandemic response.

**Bryan A**. **Liang**, **MD**, **JD**, **PhD**: BAL is Executive Director and Shapiro Distinguished Professor, Institute of Health Law Studies, California Western School of Law; Director and Professor of Anesthesiology, San Diego Center for Patient Safety, UCSD School of Medicine. His work focuses upon the systemic impact of health policy and law on patient safety, broadly construed. He combines experience working in policy contexts in Asia, the European Union, and USA with legal, medical, and economics training to formulate rational social policy promoting patient safety. He received his BS from MIT; PhD from University of Chicago; MD from Columbia; JD from Harvard.

## Pre-publication history

The pre-publication history for this paper can be accessed here:

http://www.biomedcentral.com/1472-698X/12/23/prepub
